# Supplementary material for: Content-rich biological network constructed by mining PubMed abstracts
Source: BMC Bioinformatics. 2004 Oct 8;5:147. doi: 10.1186/1471-2105-5-147 (PMC528731; doi:10.1186/1471-2105-5-147)
Supplement: Additional File 2 — The original results of the above study (non-essential files are deleted to keep the file size under the limit set by BMC bioinformatics). [file 1471-2105-5-147-S2.bz2 › chilibotAdditionalFile2/dip10/674ID10767348E11080/html/ACVRL1_ENG.html]

 


 **ACVRL1** and **ENG** 
  
Found 524 abstracts in PubMed, retrieved 10.  
 

 What does Google say? 
 PDF only 
| .edu only 

---

**Interactive relationship** (e.g. stimulation, inhibition, etc)

**Neutral relationship**- Thromboxane A 2 synthase TXAS binds to the endoplasmic reticulum membrane and catalyzes both an isomerization of prostaglandin H 2 PGH 2 to form thromboxane A 2 TXA 2 and a fragmentation of PGH 2 to form 12 hydroxy, 10 heptadecatrienoic acid HHT  [ **ENG** ]   [ **ACVRL1** ]  and malondialdehyde MDA.  Ref: 12859980 Arch Biochem Biophys, 2003
- HHT  [ **ENG** ]   [ **ACVRL1** ]  types 1 and 2 are caused by loss of function mutations in  **ENG**  and  **ACVRL1** .  Ref: 12812985 Hum Mol Genet, 2003
- The two known disease types, HHT1  [ **ENG** ]  and HHT2  [ **ACVRL1** ] , are caused by mutations in the endoglin  [ **ENG** ]   **ENG**  and ALK 1  [ **ACVRL1** ]  genes, respectively.  Ref: 12843319 J Med Genet< MedlineTA>J Med Genet, 2003
- Should asymptomatic patients with hereditary haemorrhagic telangiectasia HHT  [ **ENG** ]   [ **ACVRL1** ]  be screened for cerebral vascular malformations ? Data from 22 061 years of HHT  [ **ENG** ]   [ **ACVRL1** ]  patient life.  Ref: 12754343 J Neurol Neurosurg Psychiatry, 2003

**Non-interactive relationship** (e.g. studied together, co-existance, homology, etc.)

- Autosomal dominant hereditary haemorrhagic telangiectasia HHT  [ **ENG** ]   [ **ACVRL1** ]  is a genetically heterogeneous disease caused by mutations in at least two different loci.  Ref: 12786761 Clin Genet< MedlineTA>Clin Genet, 2003
- 1.05 to 2.99 yielded a haemorrhage rate in individuals with cerebral AV malformations of 1.4 2.0% per annum, comparable to figures in the non HHT  [ **ENG** ]   [ **ACVRL1** ]  cerebral AV malformation population.  Ref: 12754343 J Neurol Neurosurg Psychiatry, 2003
- Hereditary haemorrhagic telangiectasia HHT  [ **ENG** ]   [ **ACVRL1** ]  is a genetic vascular disorder characterised by epistaxis, telangiectases, and visceral manifestations.  Ref: 12843319 J Med Genet< MedlineTA>J Med Genet, 2003
- Substrate specificity of the alpha L arabinofuranosidase from Rhizomucor pusillus HHT  [ **ENG** ]   [ **ACVRL1** ]  1.  Ref: 12829392 Carbohydr Res, 2003
- Methods We reviewed the angiographic findings of seven patients with HHT  [ **ENG** ]   [ **ACVRL1** ] .  Ref: 12813620 Cardiovasc Intervent Radiol< MedlineTA>Cardiovasc Intervent Radiol, 2003
- A higher frequency of pulmonary arteriovenous malformations AVMs has been reported for HHT1  [ **ENG** ]  while HHT2  [ **ACVRL1** ]  is thought to be associated with a lower penetrance and milder disease manifestations.  Ref: 12843319 J Med Genet< MedlineTA>J Med Genet, 2003
- CONCLUSIONS These data contradict accepted wisdom in many countries that asymptomatic HHT  [ **ENG** ]   [ **ACVRL1** ]  patients are at a low and acceptable risk of haemorrhage.  Ref: 12754343 J Neurol Neurosurg Psychiatry, 2003
- These findings stress the need for an early diagnosis of HHT  [ **ENG** ]   [ **ACVRL1** ]  that can be useful for the early control of associated visceral involvement.  Ref: 12843319 J Med Genet< MedlineTA>J Med Genet, 2003
- The TXAS 2C5 chimera contains heme at nearly a 1 1 molar ratio and catalyzes the formation of TXA 2, MDA, and HHT  [ **ENG** ]   [ **ACVRL1** ]  at a 1 1 1 ratio, although with a reduced catalytic activity compared to wild type TXAS.  Ref: 12859980 Arch Biochem Biophys, 2003
- 12 HHT  [ **ENG** ]   [ **ACVRL1** ] , 13 78% inhibition and weaker inhibition on the formation of 12 HETE 0 49% inhibition.  Ref: 12781627 Toxicol Appl Pharmacol< MedlineTA>Toxicol Appl Pharmacol, 2003
- Haemorrhages were also six times more common in female HHT  [ **ENG** ]   [ **ACVRL1** ]  subjects 6.18.  Ref: 12754343 J Neurol Neurosurg Psychiatry, 2003
- The identification of ALK 1  [ **ACVRL1** ]  mutations in subjects with a suspected diagnosis and without clinical signs of HHT  [ **ENG** ]   [ **ACVRL1** ]  argue in favour of a molecular diagnosis.  Ref: 12843319 J Med Genet< MedlineTA>J Med Genet, 2003
- The aim of this report is to highlight the biliary manifestations of HHT  [ **ENG** ]   [ **ACVRL1** ]  and draw attention to an unusual complication of ERCP in this setting.  Ref: 12783354 Endoscopy, 2003
- METHODS Retrospective study on stroke incidence in individuals with HHT  [ **ENG** ]   [ **ACVRL1** ]  and their immediate families n = 674.  Ref: 12754343 J Neurol Neurosurg Psychiatry, 2003
- Purpose To describe the angiographic features of hepatic involvement in hereditary hemorrhagic telangiectasia HHT  [ **ENG** ]   [ **ACVRL1** ] , particularly the presence of portovenous shunts.  Ref: 12813620 Cardiovasc Intervent Radiol< MedlineTA>Cardiovasc Intervent Radiol, 2003
- The transforming growth factor beta signaling family is a key player in genetic and multifactorial diseases, including hereditary hemorrhagic telangiectasia HHT  [ **ENG** ]   [ **ACVRL1** ] , cancer, atherosclerosis and immunomodulation.  Ref: 12812985 Hum Mol Genet, 2003
- We report here a case of HHT  [ **ENG** ]   [ **ACVRL1** ]  associated with cholestasis due to common bile duct stenosis.  Ref: 12783354 Endoscopy, 2003
- Hepatobiliary manifestations of hereditary hemorrhagic telangiectasia HHT  [ **ENG** ]   [ **ACVRL1** ]  are rare, but often involve cholestasis.  Ref: 12783354 Endoscopy, 2003
- To explore the possible actions of endocrine disruptors on the autacoid synthesis in the body, we investigated the effects of nonylphenol NP, bisphenol A BPA, di n butyl phthalate DBP, benzyl n butyl phthalate BBP, and di 2 ethylhexyl phthalate DEHP on the formation of 12 lipoxygenase metabolite, 12 HETE, and cyclooxygenase metabolites, TXB 2 and 12 HHT  [ **ENG** ]   [ **ACVRL1** ] , from exogenous arachidonic acid AA in rabbit platelets.  Ref: 12781627 Toxicol Appl Pharmacol< MedlineTA>Toxicol Appl Pharmacol, 2003
- For the first time the parental germline in which a de novo HHT  [ **ENG** ]   [ **ACVRL1** ]  mutation occurred has been identified.  Ref: 12786761 Clin Genet< MedlineTA>Clin Genet, 2003
- A preliminary investigation was performed to exclude association between the predisposition and certain candidate genes including KDR kinase insert domain receptor, TEK TEK tyrosine kinase endothelial,  **ACVRL1**  activin receptor like kinase 1  [ **ACVRL1** ] ,  **ENG**  endoglin  [ **ENG** ]  and FLT4 fms like tyrosine kinase 4.  Ref: 12752572 Clin Genet, 2003
- BACKGROUND The frequency of haemorrhage in individuals with hereditary haemorrhagic telangiectasia HHT  [ **ENG** ]   [ **ACVRL1** ] , 10% of whom will have cerebral arteriovenous AV malformations, could be high enough to justify screening.  Ref: 12754343 J Neurol Neurosurg Psychiatry, 2003
- The alpha L arabinofuranosidase AF from the fungus Rhizomucor pusillus HHT  [ **ENG** ]   [ **ACVRL1** ]  1 released arabinose at appreciable rates from 1 >5 alpha L arabinofuranooligosaccharides, sugar beet arabinan and debranched arabinan.  Ref: 12829392 Carbohydr Res, 2003
- Overcorrecting for any bias towards overestimation that would be introduced in excluding non penetrant family members, cerebral haemorrhages were more than 20 times more common in male HHT  [ **ENG** ]   [ **ACVRL1** ]  subjects under the age of 45 years than in the general population standardised ratio 22.99.  Ref: 12754343 J Neurol Neurosurg Psychiatry, 2003
- 22 061 HHT  [ **ENG** ]   [ **ACVRL1** ]  patient years, specifically analysing patients under 46 years of age 17 515 patient years.  Ref: 12754343 J Neurol Neurosurg Psychiatry, 2003
- Conclusions Hepatic vascular lesions in HHT  [ **ENG** ]   [ **ACVRL1** ]  are varied, ranging from telangiectasias to large shunts between three vascular channels.  Ref: 12813620 Cardiovasc Intervent Radiol< MedlineTA>Cardiovasc Intervent Radiol, 2003
- OBJECTIVES To define the annual incidence of haemorrhagic stroke in individuals with HHT  [ **ENG** ]   [ **ACVRL1** ] .  Ref: 12754343 J Neurol Neurosurg Psychiatry, 2003
